# Facets of morbid jealousy: With an anecdote from a historical Tamil romance

**DOI:** 10.4103/0019-5545.71007

**Published:** 2010

**Authors:** O. Somasundaram

**Affiliations:** Thanigai Illam, 23^rd^ Cross, 30 Besant Nagar, Chennai, India

**Keywords:** Fiction, morbid jealousy

## Abstract

Morbid jealousy is a symptom which occurs in many psychiatric conditions. The complex emotional aspects of jealousy have been discussed by earlier authors. The clinical, cultural, social, and forensic aspects, are touched upon. Morbid jealousy is a favourite topic among novelists and dramatists. “Othello” is a classic example. This topic is covered in one of the famous historical romances of the Tamil author, Kalki.

*“O beware, my lord, of jealousy,*
*It is the green-eyed monster, which doth mock*
*The meat it feeds on.” (Othello)*
*“Jealousy is as cruel as the grave.”*
(Song of Solomon)

Morbid jealousy is not a psychiatric disorder, but a syndrome that occurs in many psychiatric conditions. Much attention has been paid to this by the French writers of earlier times. Mariet, quoted by Shepherd,[1] mentions that there are three broad sub-divisions – hyperesthetic jealousy, jealousy monomania, and delusional jealousy. Scott Buchanan as quoted by Shepherd (vide supra) expresses the view that, “in diagnosis the single symptom is radically ambiguous; it belongs to many syndromes, and it’s only legitimate interpretation demands a thorough exploration of the possible syndromes to which it belongs”.

There are any numbers of classifications of morbid jealousy, dating back to the nineteenth century French and German psychiatrists. There is a considerable overlap among them. Mairet (1908), Jaspers (1910), and Krafft-Ebbing (1891), cited by Shepherd.[1]

## Psychological aspects of jealousy

It would be appropriate to know the views of psychologists and psychiatrists about the nature of jealousy. Jealousy for Ribot was a complex state made up of pleasure, anger, and chagrin. Freud, in an article first published in 1922, writes:

“Jealousy is one of those affective states, like grief, that may be described as normal. If anyone appears to be without it, the inference is justified that it has undergone severe repression and consequently plays all the greater part in his unconscious mental life. The instances of abnormally intense jealousy met within analytic work reveal themselves as constructed of three layers. The three layers or grades of jealousy may be described as (1) competitive or normal, (2) projected, and (3) delusional jealousy.”

Normal jealousy is made up of grief, the pain at the thought of losing the loved object, and of the narcissistic wound, one’s ‘amour propre’ is hurt at the idea of losing the woman. There is also a feeling of hostility against the successful rival and a certain amount of self-criticism for losing the object. Although it is called normal jealousy, it is not always entirely rational, that is, it is not based altogether on actual situations, nor is it proportionate to the real facts or under full control of the ego. It is also to be noted that in many persons it is experienced bisexually. Thus, a jealous man may not only experience pain in regard to the loved woman and hatred toward his male rival, but he may also feel grief in regard to the unconsciously loved man and hatred toward the woman as his rival.

Projection jealousy originates in both men and women, either through their own actual unfaithfulness or through impulses to unfaithfulness that were repressed. Projection jealousy has an almost delusional character, but it is amenable to analysis, in that the patient can be made to recognize the unconscious motives to the fantasies.

The third form, delusional jealousy is more severe. It too originates on the basis of repressed impulse to infidelity, but the objects of its fantasies belong to the same sex. Delusional jealousy is, “an acidulated homosexuality and justly belongs among the classical forms of paranoia”. It is an attempt at defense against a very strong homosexual striving and in a man it may be expressed in the formula, “Indeed, I do not love him, she loves him”.[2] Later psychoanalytic workers, Fenichel and Klein added a few more details. Schiedberg as quoted by Shepherd (vide infra) has coined the term ‘Othello complex,’ which can be applied to some of these cases.

Tarrier *et al*.[3] feel that morbid jealousy is a multidimensional emotional complex relating to the unfounded suspicion of sexual and emotional rivals and a fear of losing the partner and the relationship, manifested as responses in the cognitive, affective, and behavioral domains. In this section we will describe how the morbidly jealous individual probably differs from the normal in these domains. This should form the basis of a clinical assessment.

## Cultural and social aspects

Cultural and social aspects are discussed by Mullen,[4] Mullen and Martin,[5] Bhugra.[6]

Bhugra (1993) discusses different cultural aspects of jealousy. He also points out that both males and females are prone to jealous feelings and behavior, and hence, gender does not play a substantial role. He also says that as jealousy is the product of culture, and it can vary across cultures. He also argues that individual factors such as childhood experiences, insecurity, inadequacy, dependence, and past experiences also play a key role.

## Additional psychopathology

- Preoccupation of the subject’s intrusive thoughts and suspicions about the partner’s fidelity.
- Overt behavior of seeking to confirm the suspicious preoccupations.
- Avoiding activities or situations that provoke jealousy.
- Personal distress of irritable, agitated, phobic, and suicidal ideations.
- Disruption of functioning in various spheres.
- Violence, both oral and physical, is very common in many cases of homicide; or related to this state.
- Aggression to third parties suspected to be the partner’s lover or a potential rival may occur.

## Associated psychiatric states

Shepherd in his seminal article of 1961, discusses the following:

- Morbid jealousy associated with toxic or organic cerebral disorders. Alcoholism is the most widely recognized physical association. Krafft-Ebbing is credited with making this observation in the nineteenth century. These ‘jealous drinkers’ are studied by Kolle (1932) as quoted by Shepherd (vide supra). Morbid jealousy may also be the result of dependence on stimulants like amphetamine, cocaine, and the like
- Psychiatric disorders in the elderly.
- Other cerebral disorders: presently a spate of articles refers to this association with Parkinson’s disorders
- Morbid jealousy associated with schizophrenia and delusional disorders. The ideas of infidelity occur commonly in schizophrenia and delusional states. A recent German study from Munich[7] describes the prevalence of delusional jealousy in different psychiatric disorders: The prevalence of delusional jealousy in 8134 psychiatric in-patients was 1.1%. Delusions of jealousy were most frequent in organic psychoses (7.0%), paranoid disorders (6.7%), alcohol psychosis (5.6%), and schizophrenia (2.5%); while in affective disorders, delusions of jealousy could be found in only 0.1%. As schizophrenia and affective disorders were the most common diagnoses, most patients with delusions of jealousy were schizophrenics. In schizophrenia, women were more likely to suffer from delusional jealousy, while in alcohol psychosis men were more likely to suffer from delusional jealousy.

The classification of morbid jealousy by Mullen[8] is also of relevance here.

1. Symptomatic jealousy
  Symptomatic jealousy owes its genesis to an underlying disorder of mental function, and its evolution is linked to the course of their disorder. Jealousy, when symptomatic, has the following characteristics: There is underlying disorder, usually a functional or organic psychosis, which emerges contemporaneously with or before the jealousy, with which the jealousy can be reasonably associated.
  Functional psychosis
  Schizophrenia and delusional disorders.
  Affective psychosis
  Neurotic and personality disorders.
  Toxic or Organic Cerebral Disorders (the pride of place is given to alcoholism).
  Various dementias.
2. Reactive jealousy
  - A provocation that can be reasonably related to fears about partner’s fidelity.
  - A state that sensitizes the subject to such provocations, which may be one or more of the following:
    - A personal devotion such as poor self-esteem and over-sensitivity or a superficial self-confidence covering unacknowledged vulnerability; or
    - a mental disorder, most frequently depressive; or
    - a past experience of being deceived or deserted.
  - An exaggerated psychological and behavioral response, which can on occasion, involve the development of delusional ideas.
  - A course and evolution that can be understandably related to the provoking situation and subsequent developments.

Recent clinical studies in morbid jealousy are reported by the following authors: M. Soyka, *et al*.,[7] De Silva and De Silva,[9] Kingham and Gordon.[10]

## Forensic aspects

It is well-known that morbid jealousy is responsible for a great number of crimes of violence against women. East[11] quoted by Shepherd studied 200 ‘sane’ murderers and found that 46 had killed because of jealousy, an emotion that he put alongside anger, fear, love, lust, hate, feelings of possession, and as being most frequently associated with murder. Brierly,[12] as quoted by Shepherd, made a similar study over a two-year period and found that 54 out of 760 murders in Cook County, Illinois, had occurred due to jealousy. Mowat[13] also a made a similar study of insane murderers admitted to Broadmoor over a period of 20 years and found that 12% committed their crime because of morbid jealousy. Female insane murderesses exhibited delusions of infidelity in 3.3% cases.

Somasundaram[14] studied 526 murderers of Tamil Nadu in 1968. Of the 500 male murderers 71 had killed their wives and 10 had killed their wives’ paramours. Of the six youthful offenders below 17 years old, three had killed their wives. Of the 20 murderesses four had killed their husbands, three had killed the paramours, and one had killed the co-wife.

In another study by Somasundaram[15] about the ‘Men who kill their wives,’ it was found that of 41 murderers 13 were cases of sexual jealousy and four were cases of morbid delusional jealousy. Even though there are no further studies reported from Tamil Nadu or other parts of India, it seems that delusional jealousy is fairly common in our country. It is worth recalling that a recent study from Sri Lanka, where De Silva[9] suggests that cultural aspects play a vital role in the prevalence of morbid jealousy.

## Treatment aspects

Depending on the etiology of the morbid state, treatment should be considered (anti-psychotics, anti-depressants, etc.). Psychotherapy, Cognitive Behavior Therapy, and Marital and Family Therapy should be useful.

## Morbid jealousy in literature

In literature the pride of place is given to Othello, the Moor, as subject to morbid jealousy, which results in the killing of his wife, Desdemona, and his subsequent suicide. Without going into the minutiae of the well-known Shakespearean tragedy ‘Othello’, we can mention the opinion on this character by psychiatrist Storr:[16]

Othello does not exhibit the ‘extraordinary credulousness,’ which Ernest Jones attributes to him. There are good reasons why he should trust Iago more than Desdemona, and good reasons he should be uncertain of his place in her affections. There is nothing approaching delusional jealousy in Othello’s attitude. Although Iago threatens “Othello shall go mad”, Othello shows no signs of doing so. His momentary loss of consciousness is evidence of stress, but not insanity. There are none of the other signs or symptoms that are generally found together with delusions of jealousy in people suffering from mental illness. And although Othello commits murder, and although a high proportion of murderers are suffering from mental illness, his crime is surely to be rated as a ‘crime passione,’ which, it is generally believed, is the type of murder most likely to be committed by normal people.

Another well-known reference is by Todd and Dewherst.[17]

It is somewhat surprising why our psychiatrists have not paid much attention to analyzing the characters in their own literature, comparable to their Western counterparts. Here is an attempt to delineate morbid jealousy in one of our historical novels, Kalki’s “Ponniyin Selvan”. Kalki is the pseudonym of R. Krishnamurthy (1899–1954) who was the famous editor of the Tamil weekly, “Kalki”, devoted to Indian nationalism, art and literature. He was a great personal friend of Rajaji. Among his historic novels are “Parthiban Kanavu”, “Sivagamiyin Sabatham,” and “Ponniyin Selvan”; his nationalistic novel “Alai Osai” got him the Sahitya Academy Award.

“Ponniyin Selvan” is a majestic historical romance – a masterfully woven epic of fact and conjecture set against the backdrop of Tenth Century peninsular India under the Chola Kings”, says K. Narayanan.[18]

We are concerned with some of the “dramatis personae”:

Periya Pazhuvettarayar

Lord of Pazhuvoor, Keeper of the Treasury, Chancellor of Sundara Chozhar’s Court.

Nandini Devi

His queen, the Pazhuvoor Rani.

Aditha Karikalar

Crown Prince of the Chozha empire.

Vandiyathevan

A warrior and also a friend and emissary of the Crown Prince.

Kandan Maran

Son of Lord of Kadambur.

Parthibendran

A Pallava prince, confidant of Aditha Karikalan.

Pazhuvettarayar is a great warrior, who took part in many of the Chozha campaigns to enlarge the empire under his master, Emperor Paranthaka Sundara Chozhar. He was greatly respected when he skilfully changed the rout of the Chozha army at Thakkolam into a great victory. He had taken part in 32 battles and received 96 wounds. He was considerably aged when he got infatuated with the beauty, youth, charm, and attainments of Nandini, when he sees her in the Pandiya territory and brings her to his Pazhuvoor palace in a covered palanquin, which becomes the sarcastic talk of the town. Her meeting with various handsome young men like, Vandiyathevan, Kandan Maran, and Parthibendran triggers flames of jealousy in the old man, but he controls his suspicions to a marked extent. He is magnanimous enough not to allude to his feelings, but on one occasion when Kandan Maran is accompanied by Vandiyathevan, Pazhuvettarayar arranges a hireling to stab the former in the back. Vandiyathevan carries him to his friend’s mother who is a skilled healer. Kandan Maran suspects that that he was stabbed by Vandiyathevan and their relationship is strained forever.

Nandini is the wife of the defeated Veera Pandiyan (he is in hiding). When he is chased by the victorious Karikalar, Nandini with folded hands pleads for his life. The infuriated and excited Karikalar brutally beheads the King and carries the head.

The most revealing conversation and intimate feelings between Nandini and Adithya Karikalar are brought to the fore just before the assassination of Adithar. Nandini is told that she is the step-sister of Aditha Karikalar, born to the deaf-mute mother through Sundara Chozha. She tells him the secret of her marriage to Pazhuvettarayar, which is only a ruse to enter the Chozha palace and enjoy its luxuries and carry out her intentions to wipe out the Chozha dynasty. Her various meetings with Pandiya conspirators in the palace rouses the suspicions of the old warrior, Pazhuvettarayar. Aditha Karikalar, unaware of their relationship had entertained tender feelings toward Nandini in their youth, which were discouraged by the Chozha family. Aditha recalled these happenings and suggested that they run away to a far off island. Nandini did not take to this suggestion kindly and suggests instead, that they could get rid of Pazhuvettarayar, imprison the king and ascend the throne. The infuriated Aditha Karikalar shouts at her and tells her that she wants to live with Vandiyathevan.

The intimate conversation between the two is overheard by both Vandiyathevan and Pazhuvettarayar. The latter rushes to kill Nandini with a sword. The insolvable mystery is enacted now and Aditha Karikalar is found dead in the bedroom of Nandini. It is not known who actually killed Aditha Karilkalar, although Pazhuvettarayar takes the responsibility on himself in the open court, presided over by the Chozha Emperor, and stabs himself.

The sexual jealousy felt by the old warrior toward his young beloved could be analyzed. It is clear that Pazhuvettarayar, like the other warrior Othello is not suffering from delusional psychotic jealousy. It could be considered as reactive jealousy in the words of Mullen.[8]

## Over-sensitivity and lone self esteem

The old man is advanced in years and he knows it compares unfavorably with his youthful spouse. He cannot be an attractive person with 96 wounds received in 32 campaigns (just like Julius Caesar). The reference to his age when Aditha Karikalar insultingly addresses him as ‘Thatha’ in the company of other youths infuriates him, but he suppresses his feelings.

It is worth recalling here that one of the causes of sexual jealousy mentioned in an old Buddhist text in Pali language is the old age of the husband, De Silva and De Silva.[9]

## Provoking situations and subsequent developments

There are any number of provoking situations that add to the configurations of jealousy; the presentation of the ring by Nandini to Vandiyathevan to be used as an insignia in his perambulations in the Chozha courts. Pazhuvettarayar is too great a man of honor to question Nandini directly about this happening. The confession of Nandini to Aditha Karikalar about her motive in entering the household of the old Chancellor is to wreck vengeance for the murder of her former husband Veerapandian, with no sexual motives. Throughout her stay with Pazhuvettarayar she did not allow any intimacies. Naturally this abstinence would have increased the old man’s suspicions of whether she got gratification from elsewhere. The frequent visits of the Pandiyan conspirators in the guise of magicians and astrologers would have strengthened Pazhuvettarayar’s suspicions further.

The last straw to break Pazhuvettarayar’s patience is the plan unfolded by Aditha Karikalar to elope with Nandini to a far off peaceful haven. The idea is rejected by Nandini who wants to stay back with Aditha Karikalar in Tanjavur and ascend the Chozha throne. The husband who is so far peaceful with Nandini has reached the brink of desperation and morbid jealousy leads to murderous violence[19], which has been in abeyance all these years. This leads to the murderous assault when he throws a sword at her from his hiding. Nandini is not killed in the attempt.

The last act is when Pazhuvettarayar confesses in the Chozha court about his killing of Aditha Karikalar. This great Chozha loyalist who has sworn allegiance to the Chozha family stabs himself in the presence of the royal court and subsequently dies. The domestic violence of murder and suicide so common in morbid jealousy, is thus enacted in the life of the Pazhuvettarayar.

It is not surprising that alcohol use and comorbid personality disorder are conspicuously absent in the story of the tragic warrior.

## CONCLUSION

It would be appropriate to conclude this article with an Indian touch. Bhugra,[6] mentions some unconventional marriages practiced in India in the past. The majority of the population practiced one man–one woman marriages throughout the centuries. It must be admitted that there were and are many transgressions especially by the Royal households and the upper classes. Many a time chastity is a one way affair, insisted strictly only for the woman. Men did not tolerate weakness in the women folk in spite of being broad-minded in other ways. The curse on Ahalya by her husband, the sage Gautama to be turned into a stone when she committed adultery with Lord Indra, in spite of her doubts about his identity, is an example of man’s intolerance of his spouses’ infidelity. Another legend refers to the beheading of Parasurama’s mother Renukadevi by the son at the behest of his father, sage Jamadagri, when she admired the beauty of the Gandharvas wafting in the skies.

The majority of women were chaste, exemplified by Nalayini and Kannagi.

Morbid jealousy can be prevented if the advice of Thiruvalluvar is followed:

**To woman:**

What can excel a woman

Who is rooted in chastity. (6:4)

**To man:**

A virtuous householder

Does not covet another’s wife. (14:4)

The assistance rendered by Prof. Dr. C. N. Eswari, Department of English, Guru Nanak College, Chennai, is acknowledged.
